# *Ostertagia ostertagi* exposure in dairy cows in the alpine space: implications for region-specific assessments

**DOI:** 10.1186/s13071-025-07077-3

**Published:** 2025-10-22

**Authors:** Miguel Peña-Espinoza, Manolis Lyrakis, Christian Mader, Gottfried Schoder, Simone Mitterhuemer, Barbara Hinney

**Affiliations:** 1 Institute of Parasitology, Centre of Pathobiology, Department of Biological Sciences and Pathobiology, University of Veterinary Medicine Vienna, Vienna, Austria; 2 Platform for Bioinformatics and Biostatistics, Department of Biological Sciences and Pathobiology, University of Veterinary Medicine Vienna, Vienna, Austria; 3 Animal Health Service Tyrol, Innsbruck, Austria; 4 Animal Health Service Upper Austria, Linz, Austria; 5 Institute for Veterinary Disease Control Linz, Austrian Agency for Health and Food Safety (AGES), Linz, Austria

**Keywords:** Gastrointestinal nematodes, *Ostertagia ostertagi*, Dairy cattle, Enzyme-linked immunosorbent assay, Milk production, Alpine grazing, Organic farms, Austria, Upper Austria, Tyrol

## Abstract

**Background:**

Pasture-based management of dairy cattle in temperate regions leads to infections with gastrointestinal nematodes, with *Ostertagia ostertagi* recognised as the most pathogenic species of these. However, little is known about *O. ostertagi* exposure in alpine regions. Here, we aimed to explore the *O. ostertagi* seropositivity in a large sample of dairy cows from two Austrian federal states that differ substantially in herd size, productivity and use of (alpine) pastures, and to assess associations between parasite exposure, milk yield, and farm management factors.

**Methods:**

Bulk tank milk (BTM) samples from 1241 dairy farms in the federal states of Upper Austria (*n* = 742) and Tyrol (*n* = 499), of which the dairy herds comprised a total of 25,985 adult cows, were analysed for anti-*O. ostertagi* immunoglobulin G at the end of the grazing season 2018. Anti-*Ostertagia* antibody levels in milk [measured as the optical density (OD) ratio (ODR)] from herds with known grazing were evaluated for associations with production and management data via linear models to (1) evaluate the effect of farm management practices on herd exposure to *O. ostertagi*, and (2) assess the association of *O. ostertagi* exposure and management practices with milk production.

**Results:**

Of all the investigated herds, 65.3% were positive for *O. ostertagi* infections (ODR ≥ 0.5), with a mean ODR of 0.59 (25th–75th percentile 0.42–0.75). Herds in Tyrol had higher mean BTM-ODR values (0.73; 25th–75th percentile 0.61–0.87) than those in Upper Austria (0.50; 0.38–0.63); however, alpine grazing was not associated with higher ODR values in either Austrian state. In Upper Austria, organic farming was significantly associated with higher *O. ostertagi* exposure, whereas in Tyrol, larger herd sizes were linked to increased exposure. Lower milk yield was significantly associated with decreasing cow herd size and organic farming in both federal states, but not with increasing *O. ostertagi* ODR.

**Conclusions:**

Marked differences in the exposure of Austrian dairy cattle to *O. ostertagi* are likely explained by contrasting grazing management between the investigated states and by specific characteristics of each region. No association between increased parasite exposure and milk yield was observed, highlighting the need for region-specific investigations. Small-scale and/or alpine cattle farming with predominantly dual-purpose breeds requires a tailored risk assessment to support sustainable parasite control strategies.

**Graphical Abstract:**

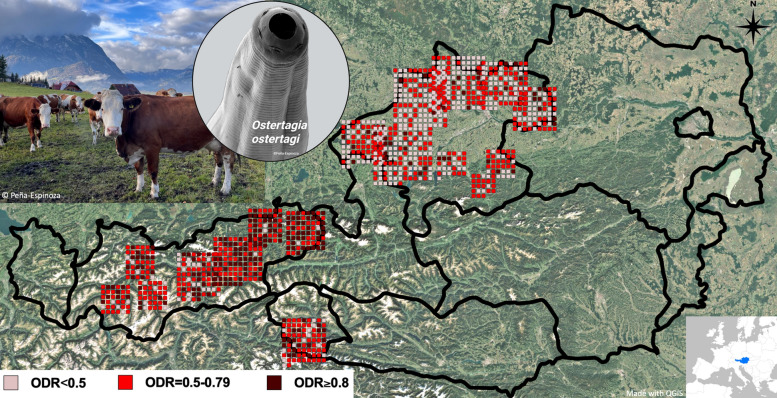

**Supplementary Information:**

The online version contains supplementary material available at 10.1186/s13071-025-07077-3.

## Background

Gastrointestinal (GI) nematodes remain some of the most prevalent pathogens in grazing livestock worldwide, with *Ostertagia ostertagi* considered the most pathogenic species infecting cattle in temperate countries, and are the cause of a substantial economic burden for the livestock industry [1–3]. Infections with GI nematodes in grazing cattle are usually considered problematic only for immune-naïve young stock, whereas adult animals are considered to develop a strong immune response that limits the clinical outcomes of this type of parasitism. However, cows exposed to pasture-borne nematode infections do not develop sterilizing immunity towards *O. ostertagi* [4]; this has been confirmed by recent post-mortem studies reporting significant worm burdens in the abomasum of adult cattle (e.g. [2, 5]). Research undertaken during the last 20 years has confirmed the negative production impacts of *O. ostertagi* infections on dairy animals [6, 7], leading to an increased awareness of the relevance of parasite monitoring also in adult cattle.

The monitoring of *O. ostertagi* in adult cattle is limited by the low nematode egg counts commonly detected in their faeces, which do not correlate with the actual worm burden [8, 9]. Additionally, the associations between parasite infections and production parameters are often highly non-linear when only parasite burdens or faecal egg counts are measured [10]. To overcome this limitation, an enzyme-linked immunosorbent assay (ELISA) was developed that evaluates the parasite exposure of dairy cows by detecting anti-*O. ostertagi* immunoglobulin G (IgG) in milk [7, 11, 12]. While this ELISA does not detect antibodies related to protective immunity, the anti-*Ostertagia* IgG levels reflect parasite exposure if measured at the end of the grazing season in regions with winter stabling [13]. Furthermore, previous studies have described a negative linear relationship between anti-*Ostertagia* IgG levels measured using this ELISA [determined as the optical density (OD) ratio (ODR)] and milk yield [14, 15]. Nevertheless, this relationship has not been consistently observed across all countries and regions [15, 16], and little is known about exposure to *Ostertagia* and its impact on milk production in dairy cattle managed in alpine regions, such as in Austria.

The dairy industry is the most significant agricultural sector in Austria, and the country has the second-highest percentage (21%) of dairy cows in the EU, after Greece, under organic farming relative to its total cattle population [17]. Further special features of Austrian dairy farms include mountain farming, the widespread use of dual-purpose cattle breeds (around 75% Simmental and only 6% Holstein), and the predominance of smallholder farms with an average dairy herd size of around 19 cows [18–20]. Milk production in Austria shows clear structural differences between the more industrialized systems in the north and east (e.g. parts of Upper Austria and Lower Austria) and farms in the mountainous west and south (e.g. Tyrol, Salzburg, Vorarlberg, Carinthia) [21]. Parasite exposure in dairy cows in Austria has been largely unexamined, with the studies that have been undertaken reporting nematode infections in a limited number of adult animals [5, 22, 23]. However, no studies have investigated regional levels of parasite exposure on a large number of farms, or the associations between parasite exposure, farm management (including alpine grazing) and productivity. Therefore, it is crucial to assess dairy cattle exposure to *O. ostertagi* locally to identify the potential geographical differences, influence of regional farm management and need for targeted interventions.

In the present study, we aimed to (1) examine the exposure levels of Austrian dairy cattle to *O. ostertagi*, (2) study associations between specific farm management factors and exposure to *O. ostertagi* in Austrian dairy cattle, (3) explore associations between *O. ostertagi* exposure and farm management factors with milk production, and (4) investigate regional differences for these findings. To capture structural differences, the study was conducted in two geographically distinct federal states: Upper Austria and Tyrol. We hypothesized that there would be higher parasite exposure on organic farms compared with conventional systems, and a negative association between exposure to *O. ostertagi* and milk production in the investigated farms. Furthermore, given the structural differences in dairy farming between Tyrol and Upper Austria, we expected significant differences between these two regions.

## Methods

### Study design

The present study is a retrospective analysis based on data collected during a previous unpublished investigation on parasite exposure as well as official national routine herd records. These data were combined to enable the analysis undertaken in this study.

### Study area

Dairy cattle farms in the Austrian federal states of Upper Austria and Tyrol were investigated. Upper Austria has the highest dairy production in Austria (one-third of the entire Austrian cattle milk production), is characterized by mainly alpine foothills and has only a minor share of alpine pastures [21, 24]. Tyrol has the highest share of alpine-grazed cattle in Austria and produces most of the country’s mountain milk, accounting for 11% of all Austrian dairy production [21], and is entirely situated in mountainous terrain (illustrated in [24]). Dairy cows and raw milk production in Upper Austria and Tyrol comprise approximately 45% of that of all of Austria [21]. Several structural differences exist between dairy farms in Upper Austria and Tyrol, including (1) herd size (average number of dairy cows per farm, Upper Austria = 26 vs. Tyrol = 12); (2) annual milk yield (Upper Austria = 7254 vs. Tyrol = 6787 kg/cow); (3) the extent of alpine pasturing (Upper Austria = 180 vs. Tyrol = 2073 alpine pastures; Upper Austria = 3% vs. Tyrol = 39% of high-altitude pastures; Upper Austria = 39 dairy cows vs. Tyrol = 31,000 dairy cows grazing on alpine pastures); and (4) the proportion of farms with a high or very high proportion of structural farming challenges such as slope, altitude, and remoteness (Upper Austria = 2% vs. Tyrol = 47%) [19, 20, 25].

### Farms, bulk tank milk samples and detection of *O. ostertagi*-specific antibodies

In a previous, unpublished, investigation, convenience samples of bulk tank milk (BTM) were collected once in 1241 dairy herds from 329 municipalities across Upper Austria (*n* = 742 farms) and Tyrol (*n* = 499 farms) after housing between October and November 2018. In Upper Austria, herds were selected from 13 of all 15 districts (10% of the ~ 7000 milk-supplying farms in the state). The two districts not represented (Linz-Land and Wels-Land) have very few milk-supplying farms and were excluded from the convenience sampling. In Tyrol, ~ 17% of the ~ 3000 herds of all nine districts were sampled. In both states, selection was based on logistical availability and was not proportional to the number of farms. BTM samples were transported in refrigerated milk tubes with preservatives (ProClin 150; Kabe Laboratories, Nümbrecht, Germany) to the Institute of Veterinary Disease Control Linz, Austrian Agency for Health and Food Safety, in Linz, Austria (samples from Tyrol) or to the laboratory of the animal health service of Upper Austria in Ried, Austria (samples from Upper Austria). Upon arrival at the laboratory, the BTM samples were stored at 5 ± 3 °C until a cream layer had formed, then milk serum was pipetted from the bottom of the tubes using steel needles via a pipetting robot (Freedom Evo; Tecan Group, Männedorf, Switzerland) into deep-well plates for storage (VWR International, Radnor, USA). BTM samples were analysed with the semi-quantitative indirect ELISA *O. ostertagi*-Ab Svanovir^®^ kit (Svanova, Uppsala, Sweden), following the manufacturer’s instructions. This ELISA kit is based on a 96-well plate containing a crude antigen from adult *O. ostertagi* that reacts with primary IgG in milk samples from exposed cattle, followed by the quantification of labelled secondary (anti-bovine) IgG using spectrophotometry; the results are expressed as ODR values [12]. The resulting ODR values are thus directly proportional to the level of anti-*O. ostertagi* IgG in the BTM sample and can be compared to the negative and positive controls provided in the kit. After incubation, the ELISA plates were read at 405 nm and the absorbance results for each sample/farm were calculated as ODR values by using the formula: ODR = (OD sample − OD negative control)/(OD positive control — OD negative control). The ODR levels obtained from the BTM samples were used to evaluate the herd exposure to *O. ostertagi*. We used the following exposure categories based on previous studies (reviewed by Charlier et al. [26]): (1) ODR < 0.5, negative farm (i.e. absence/low exposure to *O. ostertagi*); (2) ODR ≥ 0.5 and < 0.8, positive farm with exposure to* O. ostertagi*; (3) ODR ≥ 0.8, positive farm with high exposure to* O. ostertagi.*

### Milk production data and farm information

Mean milk yield/cow per farm for 2018 (mean 305-day milk production, in kilograms), calculated according to the International Committee for Animal Recording (ICAR) guidelines [27], was available for 820 farms (Upper Austria *n* = 408, Tyrol *n* = 392). These data were obtained from the respective national state control associations (Landeskontrollverband; LKV), which conduct milk recording in Austria in a standardized manner, independent of the farmer and the breeding association, and in accordance with strict national guidelines.

Farm system information (i.e. organic or conventional farm) was available for all farms. While all organic farms must provide access to pasture to adult cows during the grazing season by law [28], information on whether access to pasture was offered in conventional herds could only be inferred indirectly. Conventional farms were considered to practise grazing if voluntarily enrolled in the scheme for animal welfare and livestock grazing of the Austrian Agri-environmental Programme (Österreichisches Programm für umweltgerechte Landwirtschaft; ÖPUL) for (1) grazing (Tierschutz–Weide [29]), which involves grazing by animals for at least 120 days during the grazing season; or (2) alpine grazing (Alpung und Behirtung [29]), which requires grazing by livestock on alpine pastures for a minimum of 60 days during the grazing season. The municipalities where the cattle farms were located was known, but not the full address due to data protection. All farm management information was obtained from the Federal Ministry of Agriculture, Forestry, Regions and Water Management of Austria.

No information on the anthelmintic treatment schemes or history of parasite diagnosis for the investigated farms was available.

### Statistical analysis

#### Management variables and classification

Due to data availability, three key farm management practices could be analysed: herd size, farm system (organic/conventional farming) and alpine grazing. Because farms classified as having “non-grazing herds” might still graze their cattle, statistical analyses were restricted to data from farms known to allow grazing (i.e. organic or enrolled in the ÖPUL-grazing/alpine grazing schemes), thereby avoiding misclassifications in the analysis.

#### Control for confounding effects and assessment of state-specific effects

The federal states Tyrol and Upper Austria are situated in regions that show divergent climatic conditions and differ in farm structure, overall milk production and the degree of natural structural farming challenges (see above). Since these two regions are not directly comparable, the following models took the federal state into account to control for confounding effects and to identify differences in regard to farm management or parasite-exposure effects within each state. Given the pronounced differences in the latter, it was methodologically necessary to test for potential state-specific effects by including interaction terms between federal state and the other predictors, thereby avoiding spurious overall effects that might have arisen if differences had been driven solely by one state or if opposing trends had been masked. For these reasons, only the interaction effects and not the main effects were analysed.

### Model development

All statistical analyses were performed in R version 4.1.2 [30]. For visualization of the results we used ggplot2 (function ggplot, R package ggplot2, version 3.4.1.) [31].

#### Model 1: effect of farm management practices on herd exposure to *O. ostertagi*

Farm management effects on parasite exposure were evaluated via a linear model (function lm) with data from 642 farms with BTM ELISA results (ODRs), known grazing and complete farm information (see above). BTM farm ODR was fitted as a numerical response. The interactions between federal state (Tyrol/Upper Austria) and farming system (organic/conventional), federal state and alpine grazing (yes/no) and federal state and log10-transformed cow herd size were fitted as fixed effects. Pairwise contrasts between the levels of a categorical predictor for each level of the other categorical predictor present in the interaction were conducted via the estimated marginal means (function emmeans, R package emmeans, version 1.8.5., options pairwise ~ predictor1 | predictor2, adjust = none [32]) and corrected for multiple testing within the predictor via the Bonferroni-Holm method (function p.adjust). Similarly, effects of log10-transformed cow herd size were evaluated for each state (functions emtrends and test, R package emmeans, options in emtrends pairwise ~ state, var = log10(Herd_Size), adjust = none).

#### Model 2: effect of herd exposure to *O. ostertagi* and farm management on milk production

To evaluate the effect of parasite exposure and farm management on milk yield, a linear model was designed using data from farms with known grazing that had complete milk production, cow herd size information and ODR (*n* = 489 farms). The final model included mean milk yield per cow (mean 305-day milk in 2018, in tons) as the numerical response variable. The interactions between federal state (Tyrol/Upper Austria) and farming system (organic/conventional), federal state and alpine grazing (yes/no), federal state and log10-transformed cow herd size and federal state and ODR (numerical or categorical with three levels) were fitted as fixed effects. Contrasts and slopes evaluation was conducted as described above. Milk yield differences/cow per day were calculated by converting the estimated contrasts/slopes into kilograms and dividing by 305.

Multicollinearity was evaluated via variance-inflation factors (function vif, R package car, version 3.1–1. [33]). Assumptions about the residuals in regards to the aforementioned linear models were visually evaluated, with no obvious violations observed. Significance for all analyses was declared at an alpha cut-off of 5% (*p* < 0.05) after multiple testing correction.

## Results

### Farm information

BTM was obtained from 1241 dairy farms distributed between Upper Austria and Tyrol; their dairy herds comprised a total of 25,985 adult cows [mean herd size (minimum–maximum) 21.49 (1–174)]. Of all the investigated farms, 86.9% were conventional and 13.1% organic. Mean cow herd size, milk yield and farm management practices between organic and conventional farms in each federal state are presented in Table 1. All organic farms in both states provided their cows with access to pastures, whereas grazing by cows was practised by at least 90.4% of the conventional farms in Tyrol, compared with at least 13.7% of the conventional farms with known grazing in Upper Austria. Furthermore, 85.2% of the cattle farms in Tyrol practised alpine grazing, compared to < 10% of farms in Upper Austria (Table 1).

**Table 1:** Summary of production and management information for the sampled dairy cattle farms used to investigate the exposure of their dairy herds to *Ostertagia ostertagi* as assessed in bulk tank milk (BTM) samples from two federal states in Austria (Tyrol and Upper Austria) collected at the end of the grazing season 2018 (*n* = 1241)

Farms investigated in Tyrol and Upper Austria	Tyrol (*n* = 499)		Upper Austria (*n* = 742)	
	Conventional (*n* = 450)	Organic (*n* = 49)	Conventional (*n* = 629)	Organic (*n* = 113)
Arithmetic mean cow herd size (min.–max.)^a^	15 (1—111)^c^	12 (2—55)	26 (2—174)	27 (4—118)
Arithmetic mean milk yield (kg/cow per year (± SD)^a^	7133 (± 1512)	6482 (± 1443)	7937 (± 1473)	6840 (± 935.6)
Number of farms with known grazing by cattle^b^	407/450 (90.4%)	49/49 (100%)	86/629 (13.7%)	113/113 (100%)
Number of farms with alpine grazing by cattle^b^	381/450 (84.7%)	44/49 (89.8%)	16/629 (2.5%)	7/113 (6.2%)

*min*. Minimum,* max*. maximum

^a^The information was not available for all the farms from which BTM samples were obtained

^b^The information was available for all of the sampled farms. Grazing by cattle and alpine grazing by cattle were inferred for conventional farms based on their voluntary enrolment in the relevant scheme(s) of the Austrian Agri-environmental Programme (Österreichisches Programm für umweltgerechte Landwirtschaft; ÖPUL)

^c^Where *n* = 1, the BTM sample corresponds to the milk of a single cow

### Exposure to *O. ostertagi*

**Fig. 1:**
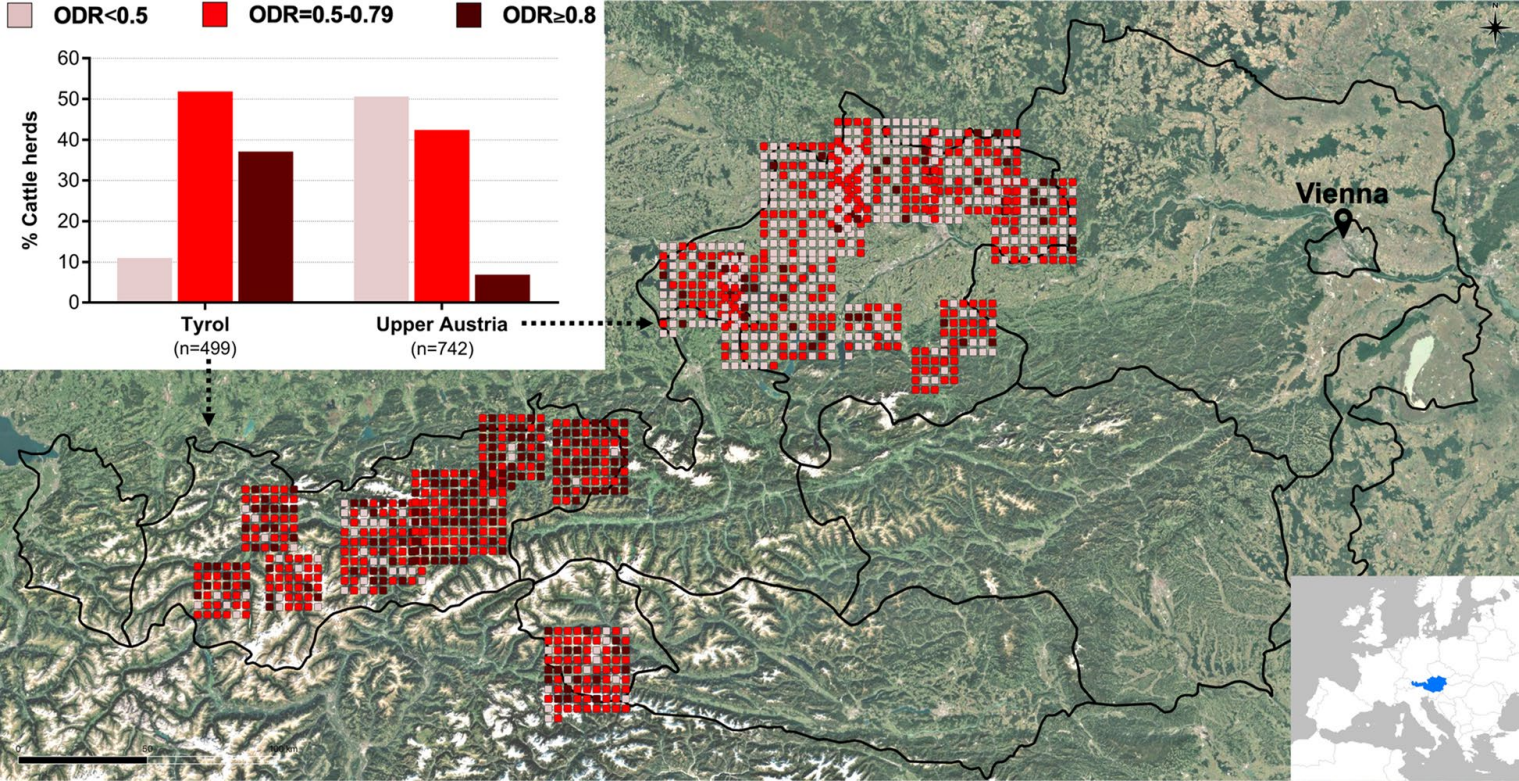
General distribution of dairy cattle farms investigated for anti-*Ostertagia ostertagi* immunoglobulin G (IgG) levels in bulk tank milk (BTM) from two federal states of Austria, Tyrol (*n* = 499) and Upper Austria (*n* = 742). Each square in the map symbolises the individual optical density ratio (ODR) category identified per farm: negative farms (ODR < 0.5), *Ostertagia*-positive farms with potential production losses (ODR 0.5—0.79), and *Ostertagia*-positive farms with potentially significant production losses (ODR ≥ 0.8). The positions of the farms on the map do not correspond to their actual locations (which were unknown to the authors) and only represent the approximate distribution of the farms within each state. Points that lie outside the country/state boundaries are visual effects due to the increased size of each point relative to the scale of the map in GIS software (map made with QGIS; www.qgis.org)

A summary of the ODR levels detected among all sampled dairy farms is presented in Table 2, and the distribution of ODR categories detected in individual farms across Upper Austria and Tyrol is shown in Fig. 1. Considering all sampled herds (*n* = 1241), 65.3% were positive for *O. ostertagi* infections (ODR ≥ 0.5), with a mean ODR of 0.59 (25th–75th percentile 0.42–0.75) across all farms. Overall, herds in Tyrol had higher mean BTM-ODR values (0.73; 25th-75th percentile 0.61—0.87) than those in Upper Austria (0.50; 0.38—0.63).

**Table 2:** Exposure to *Ostertagia ostertagi* on dairy cattle farms (*n* = 1241) in two federal states in Austria (Tyrol and Upper Austria) measured by detection of anti-*O. ostertagi* immunoglobulin G (IgG) antibodies in BTM at the end of the grazing season 2018

Farms investigated in Tyrol and Upper Austria	Tyrol (*n* = 499)		Upper Austria (*n* = 742)	
	Conventional (*n* = 450)	Organic (*n* = 49)	Conventional (*n* = 629)	Organic (*n* = 113)
Arithmetic mean ODR (min.–max.)	0.73 (0.12—1.18)	0.77 (0.26—1.14)	0.47 (0.00—1.19)	0.65 (0.02—1.00)
25—75% Percentile ODR	0.61—0.86	0.68—0.87	0.33—0.61	0.56—0.75
Farms positive for *O. ostertagi* exposure (ODR ≥ 0.5)	399/450 (88.7%)	45/49 (91.8%)	268/629 (42.6%)	98/113 (86.7%)
Positive farms with high *O. ostertagi* exposure (ODR ≥ 0.8)	165/399 (41.4%)	20/45 (44.4%)	35/268 (13.1%)	16/98 (16.3%)

*ODR* Optical density ratio (*Ostertagia ostertagi* BTM enzyme-linked immunosorbent assay result)

### Effects of farm management practices on herd exposure to *O. ostertagi*

**Fig. 2:**
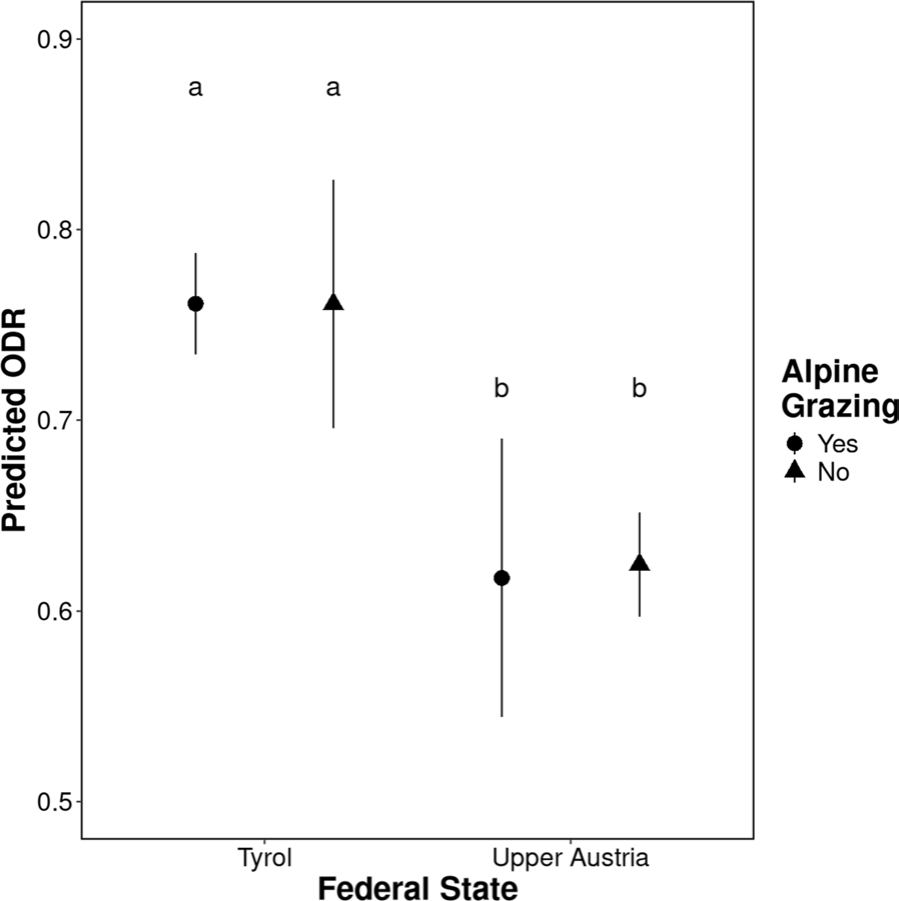
Alpine grazing effect on exposure to *Ostertagia ostertagi* measured as ODR for anti-*Ostertagia ostertagi* IgG levels (response variable) in BTM from dairy cattle farms with known grazing (*n* = 642) in two federal states in Austria. Estimated marginal means (predicted ODR) and their 95% confidence intervals, inferred by the linear model, are presented. Pairwise comparisons were corrected for multiple testing via the Bonferroni-Holm method. Different letters correspond to significant differences at an alpha cut-off of 5% (*p* < 0.05) after multiple testing correction. For abbreviations, see Fig. 1

**Fig. 3:**
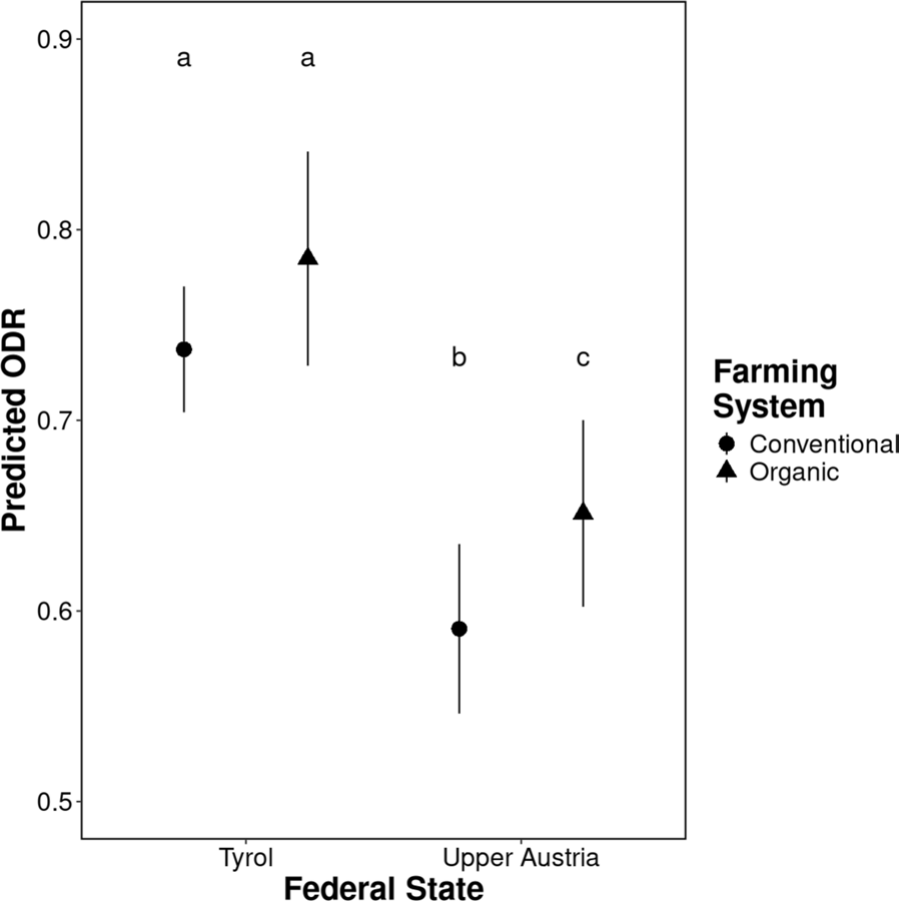
Farming system effect on exposure to *Ostertagia ostertagi* measured as ODR for anti-*Ostertagia ostertagi* IgG levels (response variable) in BTM from dairy cattle farms with known grazing (*n* = 642) in two federal states in Austria. Estimated marginal means (predicted ODR) and their 95% confidence intervals, inferred by the linear model, are presented. Pairwise comparisons were corrected for multiple testing via the Bonferroni-Holm method. Different letters correspond to significant differences at an alpha cut-off of 5% (*p* < 0.05) after multiple testing correction. For abbreviations, see Fig. 1

Results of the first model, which tested the effects of farm management practices on parasite exposure of herds with known grazing (*n* = 642), are presented in Table 3. Alpine grazing had no effect on ODR levels in either of the investigated regions (Fig. 2; Table 3). Farming system (organic/conventional) had a significant effect on parasite exposure only in Upper Austria, with cows on conventional farms with grazing having lower ODR levels than organic herds (*p* = 0.023; Fig. 3). Cow herd size had a significant effect on exposure to *O. ostertagi* only in Tyrolean farms, where an increase in herd size resulted in a significant increase of ODR levels (*p* < 0.0001; Table 3). Across all farm management practices, higher ODR levels were observed in Tyrol compared to Upper Austria (*p* < 0.0001; Table 3).

**Table 3:** Contrasts and slopes from the linear model investigating the effects of farm management practices on exposure to *Ostertagia ostertagi* measured as ODR for anti-*Ostertagia ostertagi* IgG levels (response variable) in BTM from dairy cattle farms with known grazing (*n* = 642) in two federal states in Austria, Tyrol and Upper Austria

Contrasts/slopes	Interacting levels	Estimate/trend	SE	*p*-value
Alpine grazing no—yes	Tyrol	0.004	0.032	1.000
	Upper Austria	0.002	0.039	1.000
Tyrol—Upper Austria	Alpine grazing no	0.158	0.036	< 0.0001****
	Alpine grazing yes	0.156	0.039	< 0.0001****
Conventional—organic	Tyrol	-0.044	0.026	0.092
	Upper Austria	-0.064	0.025	0.023*
Tyrol—Upper Austria	Conventional	0.167	0.028	< 0.0001****
	Organic	0.147	0.038	< 0.0001****
Cow herd size (log10 cows/farm)	Tyrol	0.110	0.022	< 0.0001****
	Upper Austria	-0.073	0.044	0.097

*p*-values after multiple testing correction are presented (Bonferroni–Holm method). For abbreviations, see Tables 1 and 2

### Effects of herd exposure to *O. ostertagi* and farm management practices on milk production

Results of the second model, which tested the effects of parasite exposure and farm management practices on milk production with herds of known grazing and production data (*n* = 489), are presented in Table 4. Two predictors had significant effects on annual milk yield in both federal states: cow herd size and farming system (Table 4; Suppl. Table 1). An increasing number of cows per herd was associated with higher farm milk production, with a unit log10 increase of cows (e.g. 10–100) leading to a 5.6 kg higher mean milk yield per animal per day in Tyrol (*p* < 0.0001) and 4.5 in Upper Austria (*p* = 0.013). Organic farms were associated with lower daily milk yield per cow compared with conventional herds, with a mean reduced milk production of 1.7 kg in Tyrol (*p* = 0.017) and 3.0 kg in Upper Austria (*p* < 0.0001). No significant effect of *Ostertagia* exposure on milk yield was observed (for the numerical as well as the categorical variables). Furthermore, access to alpine grazing had no effect on milk yield, and no significant differences between Tyrol and Upper Austria were observed for any of these variables (Table 4; Suppl. Table 1).

**Table 4:** Contrasts and slopes from the linear model investigating the effects of *Ostertagia ostertagi* exposure (ODR, numerical) and farm management practices on milk yield per cow (mean 305-day milk production in 2018, in tons) on cattle farms with known grazing and milk production data (*n* = 489) from two federal states in Austria, Tyrol and Upper Austria

Contrasts/slopes	Interacting levels	Estimate/trend	SE	*p*-value
ODR	Tyrol	− 0.551	0.402	0.341
	Upper Austria	0.079	0.768	0.918
Alpine grazing no—yes	Tyrol	0.153	0.306	1.000
	Upper Austria	− 0.140	0.359	1.000
Tyrol—Upper Austria	Alpine grazing no	0.338	0.356	0.685
	Alpine grazing yes	0.045	0.364	0.902
Conventional—organic	Tyrol	0.511	0.214	0.017*
	Upper Austria	0.927	0.265	< 0.0001****
Tyrol—Upper Austria	Conventional	− 0.016	0.284	0.956
	Organic	0.399	0.355	0.521
Cow herd size (log10 cows/farm)	Tyrol	1.697	0.209	< 0.0001****
	Upper Austria	1.380	0.556	0.013*

*p*-values after multiple testing correction are presented (Bonferroni–Holm method). For abbreviations, see Tables 1 and 2

## Discussion

This study represents an initial approach to evaluating exposure to *O. ostertagi* in Austrian dairy cows based on a large number of herds. Higher parasite exposure on dairy farms in alpine regions (Tyrol) was identified. Significant associations between higher ODR levels and an increasing number of dairy cows (Tyrol) and organic farming (Upper Austria) were observed. No significant association between increasing ODR and milk production was identified. Several biological and farm management factors are likely to have contributed to the observed results, as discussed below.

Overall, mean ODR levels detected across all sampled Austrian herds showed a higher prevalence of *Ostertagia* infections (ODR ≥ 0.5) compared with recent studies employing data collected over a large scale in Germany and Italy that used the same BTM ELISA [34–36], but lower prevalences than those reported for Switzerland [16] and in earlier studies undertaken in Belgium, Ireland and the UK [15, 37].

Here, marked differences in ODR levels were observed between BTM samples from different federal states and farming systems within Austria, which mainly reflected the distinct effect of pasture grazing. While the vast majority of the sampled Tyrolean farms (conventional and organic) allowed their cows access to pasture and had the highest mean ODR values detected, conventional herds in Upper Austria had the least access to pasture grazing and, not surprisingly, the lowest mean ODR levels. However, as access of the cows to pastures on the sampled farms could only be inferred from the voluntary registration of the farms in a national agri-environmental programme, these results have to be interpreted with caution.

Furthermore, details on the grazing age groups, time of grazing per day and the length of the grazing season or anthelmintic treatment were unknown, but are factors that can critically affect parasite exposure in dairy cattle (e.g. [37, 38]). On average, cattle have longer grazing seasons in Tyrol than in Upper Austria [19], which might be a major reason for the higher *Ostertagia* exposure in Tyrol. Tyrolean farms also had higher *Ostertagia* exposure as herd size increased, which contrasts with findings from other studies on large-scale dairy farms, e.g. in Belgium and UK [37], but are in accordance with the results of a Swiss report on a similar husbandry system [16]. These results may indicate that higher stocking densities of larger herds in mountain farming systems can lead to increased parasite exposure in these regions.

In Upper Austria, the higher *Ostertagia* exposure of herds on organic farms in comparison with those on conventional farms is likely due to the greater reliance on pasture grazing and restricted use of anthelmintic treatments of the former. Similar findings were reported in a randomized Swedish study [39]. Considering the high proportion of organic farming in Austria, and the planned increase of organic farmland in the EU by 2030 [40], our results indicate that further research on current parasite exposure in organic herds is warranted.

Interestingly, this difference between farming systems was not observed in Tyrol, with both conventional and organic farms having similar levels of anti-*Ostertagia* antibodies in their BTM samples, despite the expected higher exposure to pasture-borne nematodes following the legally limited use of anthelmintics and the longer grazing season in organic systems there [28]. The similar risk of *Ostertagia* infections between conventional and organic herds in Tyrol may indicate that the grazing management practices of the organic and conventional dairy farms, including the grazing of alpine pastures (see below), are comparable there, and that this needs to be studied in detail.

No significant effect of alpine grazing on *Ostertagia* exposure in dairy cattle was observed in any of the studied regions. However, classifying farms simply as using “alpine grazing” may not sufficiently capture the potential effects of this practice. Notably, Tyrolean herds, for which around 85% of the sampled farms practised alpine grazing, showed significantly higher parasite levels compared to herds in Upper Austria, where only 4% of farms are managed on mountain pastures. This suggests that alpine grazing could indeed be a risk factor for *Ostertagia* exposure, but more in depth epidemiological studies of GI nematodes in mountain grasslands are needed to confirm this.

High exposure to *O. ostertagi* in dairy cows evaluated using the BTM ELISA has been associated with lower milk yield when infections are subclinical [26]. In the present study, no significant association between increased *Ostertagia* exposure and reduced milk production was observed. Interestingly, no association between milk production and *Ostertagia* exposure was detected in Swiss dairy herds with a similar farming structure [16]. Similarly, recent studies in Germany concluded that *Ostertagia* seropositivity in dual-purpose breeds was not associated with milk production penalties, potentially due to their higher resilience to infections [36, 41]. The data obtained in these studies thus suggest that parasite control recommendations developed from surveys undertaken in regions with conventional large-scale farms cannot be simply applied to smallholder farms in mountain regions with dual-purpose breeds, many types of structural farming challenges and under organic farming with a lower level of baseline production, where milk output is influenced by multiple, non-optimised management factors. In such systems, the potential benefits of parasite control may be less evident than in high-yielding, intensively managed herds.

However, certain factors in the study design might have contributed to the absence of a detectable relationship between parasite exposure and milk yield. One possible factor may be the available production data. Milk yield was represented by the 305-day average per farm, rather than calculated from individual cow records (as e.g. in Charlier et al. [14]). Thus, while milk from all the cows on a farm was included in the sampled BTM, milk from some cows was excluded from the 305-day yield data. For example, milk from a primiparous cow with a high *Ostertagia* antibody titre but lactation shorter than the period of 305 days would have been included in the sampled BTM but not for the yield estimate calculation, potentially introducing bias into the analysis.

Additionally, no data on antiparasitic treatment protocols were available for the farms included in this study, and given the influence of multiple factors on milk production, it is important to note that observational studies that describe associations cannot demonstrate causality. To examine if treatment against *Ostertagia* would influence productivity in small holder farms in alpine regions, an intervention study with anthelmintic treatment would thus be required. Although an intervention study conducted under similar farming conditions in South Tyrol, Italy, reported an effect of treatment on milk yield [42], study-specific constraints (e.g. significant baseline differences in animal age between control and treatment groups) indicate that the treatment effect should be reassessed for Austrian farming conditions.

Additionally, economic studies indicate that maximization of production through helminth control tends to be more profitable for farms with high technical efficiency [43]. Given that technical efficiency on many Austrian farms is rather low [44], it is important to investigate the benefit of anthelmintic treatment and/other parasite control interventions within a broader economic and environmental context.

As also observed in previous studies [45, 46], farm size and organic farming were significantly associated with milk production. A higher number of cows per farm was associated with enhanced annual milk production per animal, whereas organic systems were associated with lower milk yield per animal. Organic dairy farms in the EU produce 8–33% less milk than their conventional counterparts [47] due to, among other factors, their lower use of external inputs such as high-energy feed concentrates and greater reliance on forage feeds (which have a lower energy content) in the animals’ diets [46]. Moreover, the potential impact of parasite infections on the yield gap between organic and conventional dairy herds warrants further research.

One limitation of our study was the selection of farms for sampling of BTM samples based on non-random convenience sampling without proportional allocation by herd size. As a consequence, the sample is not representative, and prevalence estimates could be biased. However, the inclusion of herds from almost all the districts of the states ensured broad geographical and climatic coverage. Mean herd sizes were similar to the annual averages or slightly higher than them (e.g. about three cows more on Tyrolean conventional farms).

A further drawback was the lack of information on anthelmintic treatments on the farms, which means that lower *Ostertagia* exposure in the investigated herds could have been the result of (effective) anthelmintic treatments during the studied season. In addition, the measurement of anti-*Ostertagia* antibody levels in BTM samples is influenced by several factors, such as the number and relative seropositivity of cows whose milk is included in the BTM, the high variability in antibody titres between animals in the same herd, the stage of lactation, the age and the milk yield of the animals, and the dilution of antibodies from individual animals, as well as cross-reactivity of the BTM ELISA *O. ostertagi* crude antigen with antibodies to other cattle helminths such as *Cooperia oncophora* and *Fasciola hepatica* [13, 37, 48, 49].

Also, in our dataset, some BTM samples originated from very small herds (from one to five lactating cows), which are common in the study regions, but are not directly comparable to BTM from larger herds that were used to validate the interpretation of BTM *Ostertagia* antibody levels in other studies. Therefore, our results can only be interpreted as an initial approach to estimating the general exposure of dairy cattle to *O. ostertagi* in Austria and not as conclusive of the presence of the parasite in the animals whose milk was included in the investigated BTM samples.

Moreover, ODR levels in milk samples are known to vary during the grazing season, and further studies focusing on the evaluation of ODR levels of milk from individual animals sampled at different time points during the grazing season, including their correlation with BTM-ODR levels, may help to further elucidate the current dynamics of *Ostertagia* infections in Austrian dairy cattle. Nevertheless, our results can be interpreted as a proxy for exposure to, and hence pasture contamination with, the parasite *O. ostertagi* in the investigated Austrian herds and regions.

Pasture access for dairy cattle is promoted in Austria and other European countries, with the aim of increasing animal welfare and sustainability [50, 51]. Infections with GI nematodes in grazing cattle are expected to remain a key challenge for animal health that requires the implementation of sustainable helminth control approaches. Integrating BTM ODR with grazing management indicators may improve targeted treatment decisions and should be validated under Austrian conditions, particularly in the case of more exposed systems such as Tyrolean and organic farms.

## Conclusions

Most of the examined Austrian dairy herds were positive for *O. ostertagi* infections, albeit with marked differences between the investigated federal states and with specific characteristics within each region. Between regions, contrasting grazing management of dairy herds in the investigated Austrian states likely explained the observed differences in parasite exposure. In Upper Austria, organic farming was significantly associated with higher *O. ostertagi* exposure, whereas in Tyrol larger herd sizes were linked with more parasite infections. Our study highlights the need for region-specific investigations and tailored parasite risk assessments, as generalized recommendations from other regions and countries cannot be directly applied to the regions studied here. This research is necessary to develop sustainable parasite control strategies relevant to local farming communities and to improve the accuracy of epidemiological and economic modelling.

## Supplementary Information

Supplementary file 1.

## Data Availability

Data supporting the main conclusions of this study are included in the manuscript.
