# Supplementary material for: Ostertagia ostertagi exposure in dairy cows in the alpine space: implications for region-specific assessments
Source: Parasit Vectors. 2025 Oct 22;18:425. doi: 10.1186/s13071-025-07077-3 (PMC12542466; doi:10.1186/s13071-025-07077-3)
Supplement: Supplementary file 1 [file 13071_2025_7077_MOESM1_ESM.docx]

**Supplementary Table 1.** Contrasts and slopes from the linear model investigating the effects of *Ostertagia ostertagi* exposure (ODR, categorized into **three groups**) and farm management practices on milk yield per cow (mean 305-day milk production in 2018, in tons) in cattle farms with known grazing and milk production data (n=489) from two federal states in Austria. *p*-values after multiple testing correction are presented (Bonferroni–Holm method).

| Contrasts/Slopes | Interacting Levels | Estimate/Trend | Standard Error | *p*-value |
| --- | --- | --- | --- | --- |
| ODR low – ODR intermediate* | Tyrol | -0.034 | 0.234 | 1.000 |
|  | Upper Austria | -0.090 | 0.299 | 1.000 |
| ODR low – ODR high* | Tyrol | 0.246 | 0.247 | 1.000 |
|  | Upper Austria | 0.506 | 0.482 | 1.000 |
| ODR intermediate – ODR high* | Tyrol | 0.280 | 0.145 | 0.327 |
|  | Upper Austria | 0.597 | 0.430 | 0.828 |
| Tyrol – Upper Austria | ODR low* | 0.235 | 0.413 | 1.000 |
|  | ODR intermediate* | 0.179 | 0.287 | 1.000 |
|  | ODR high* | 0.496 | 0.452 | 0.821 |
| Alpine grazing No - Yes | Tyrol | 0.149 | 0.306 | 1.000 |
|  | Upper Austria | -0.168 | 0.359 | 1.000 |
| Tyrol – Upper Austria | Alpine grazing No | 0.462 | 0.368 | 0.420 |
|  | Alpine grazing Yes | 0.144 | 0.371 | 0.697 |
| Conventional - Organic | Tyrol | 0.516 | 0.213 | **0.016** |
|  | Upper Austria | 0.893 | 0.266 | **<0.0001** |
| Tyrol – Upper Austria | Conventional | 0.114 | 0.291 | 0.695 |
|  | Organic | 0.492 | 0.368 | 0.363 |
| Cow herd size (log10-cows/farm) | Tyrol | 1.695 | 0.211 | **<0.0001** |
|  | Upper Austria | 1.201 | 0.564 | **0.034** |

* ODR low – ODR < 0.5, ODR intermediate - 0.5 ≤ ODR < 0.8, ODR high – ODR ≥ 0.8
